# Coaxial Jet Mixing for Pharmaceutical Nanocarrier Production: Experimental Analysis and Mechanistic Modeling

**DOI:** 10.3390/pharmaceutics18040507

**Published:** 2026-04-20

**Authors:** Diego Caccavo, Raffaella De Piano, Francesca Landi, Gaetano Lamberti, Anna Angela Barba

**Affiliations:** 1 Department of Industrial Engineering, University of Salerno, 84084 Fisciano, Italy; dcaccavo@unisa.it (D.C.); rdepiano@unisa.it (R.D.P.); francesca.landi96@gmail.com (F.L.); 2 Eng4Life Srl, Via Circumvallazione, 39, 83100 Avellino, Italy; aabarba@unisa.it; 3 EST Srl, Academic Spin-Off, Via Circumvallazione, 39, 83100 Avellino, Italy; 4 Department of Pharmacy, University of Salerno, 84084 Fisciano, Italy

**Keywords:** coaxial jet mixer, antisolvent precipitation, nanoliposomes, population balance modeling, computational fluid dynamics

## Abstract

**Background/Objectives:** This study addresses the need for scalable and predictive strategies linking mixing conditions to nanocarrier properties by developing and analyzing a coaxial jet antisolvent process for the continuous production of pharmaceutical nanocarriers. **Methods:** A single experimental platform was used to generate both curcumin-based nanoparticles and nanoliposomes, enabling direct comparison of how mixing regime and formulation variables influence product characteristics. **Results:** Fluid-dynamic behavior was first characterized using tracer and micromixing experiments, revealing a strong dependence of mixing time on flow conditions, with characteristic mixing times decreasing from >1000 ms under laminar conditions to approximately 10–30 ms in turbulent regimes. Nanoparticles and liposomes obtained under optimized conditions exhibited mean sizes in the range of 120–250 nm, with polydispersity indices typically below 0.2 under optimized turbulent conditions. To rationalize these observations, a computational framework was implemented, combining Reynolds-averaged computational fluid dynamics with a population balance formulation solved by the method of moments. The model provided spatially resolved insight into solvent exchange, supersaturation development, and nucleation–growth dynamics, showing good agreement with experimental trends and capturing the effect of mixing conditions on particle size across different regimes. **Conclusions:** Although simplified, the modeling approach establishes the basis for future extensions toward full population-balance distribution simulations capable of predicting complete particle size distributions, highlighting the ability of the coaxial jet mixer to control supersaturation and particle formation through tunable hydrodynamic conditions. This capability makes the system particularly attractive compared to conventional batch or less controllable mixing technologies, enabling a more rational and scalable design of pharmaceutical nanocarriers, with good encapsulation performance as discussed in the main text.

## 1. Introduction

The pharmaceutical pipeline today contains a high number of lipophilic active pharmaceutical ingredients (APIs) whose in vitro potency is not matched by in vivo efficacy because of extremely limited aqueous solubility, which translates directly into low bioavailability [1,2]. Poor solubility reduces dissolution rate in gastrointestinal fluids, limits the saturable fraction available for absorption, exacerbates first-pass metabolism and magnifies inter-subject pharmacokinetic variability; all these effects combine to produce subtherapeutic plasma exposures, the need for high oral doses, and frequent clinical failure or requirement for complex dosing regimens [3,4,5]. Moreover, many lipophilic APIs are chemically fragile (susceptible to oxidation, hydrolysis, or photodegradation) and may undergo pre-absorptive loss, further lowering the effective bioavailable dose [2,3,6]. Therefore, increasing bioavailability is not simply a formulation convenience but a critical enabling step that converts active molecules into deliverable medicines. Improving bioavailability reduces dose, mitigates toxicity associated with high systemic exposure, and supports reproducible therapeutic windows across patient populations [1,7]. Because fundamental drivers of bioavailability include dissolution rate, surface area, and barrier permeation, formulation work must address these parameters simultaneously—by controlling particle size and morphology, protecting labile actives from degradation, and selecting excipients that promote absorption [5,6,8]. Industry also demands methods that are scalable, compliant with solvent-residue and GMP constraints, and that deliver narrow size distributions (low PDI) for predictable behavior; consequently, there is ongoing emphasis on technologies that can reproducibly reduce particle dimensions to the nanoscale without untenable process complexity or solvent burdens [9,10].

A wide spectrum of delivery systems has been developed to overcome solubility-limited bioavailability: lipid vehicles (liposomes, solid lipid nanoparticles, nanostructured lipid carriers), polymeric nanoparticles and micelles, self-emulsifying drug delivery systems (SEDDS/SMEDDS), and nano-emulsions and nanosuspensions [3,5,11,12,13]. All these systems share a common mechanistic rationale—increase the apparent solubility and interfacial area available for mass transfer, protect the API from chemical attack, and in some platforms enable transcellular or lymphatic transport routes that bypass first-pass metabolism [14,15]. Each of these platforms addresses the solubility problem in a distinct way: lipid systems solubilize APIs in hydrophobic compartments, polymeric micelles encapsulate them in cores shielded by hydrophilic coronas, and self-emulsifying systems generate fine oil droplets in situ upon contact with aqueous fluids. Despite their diversity, these systems share a key principle: smaller particles or droplet size generally correlate with improved dissolution, absorption, and bioavailability [14,16]. Then, among the formulation variables, particle (or droplet) size repeatedly emerges as the dominant, quantitative determinant of performance: shrinking a particle from micron to sub-micron and further to the true nano-regime dramatically increases surface area available for dissolution, reduces diffusion distances for API release, and alters interaction with mucus and epithelial layers [16,17]. Empirically, nanoparticles (<200 nm) typically show higher dissolution rates and, for many lipophilic molecules, greater oral bioavailability than microparticles (hundreds of nm to a few µm) or larger crystals, provided that aggregation is controlled and stabilizers do not impair absorption. However, very small sizes can raise stability and manufacturability challenges: increased propensity to Ostwald ripening, higher requirements on stabilizer selection, and potential for altered biodistribution [5,18]. Hence, a successful formulation strategy optimizes not only mean size but also polydispersity, surface composition (PEGylation, surfactant layer), and physical state (amorphous vs. crystalline), because these jointly shape dissolution kinetics and biological uptake. For these reasons, technologies that deliver both reliable nanosizing and narrow PDI are of primary interest to industry and to regulators aiming at consistent product performance [5,19].

Technological approaches to nanoparticle production can be broadly categorized as top-down, bottom-up, or hybrid methods [20,21]. Top-down approaches such as wet milling, high-pressure homogenization, and microfluidization physically break down larger crystals into nanosized particles. These methods are robust, already deployed at an industrial scale, and often result in nanosuspensions with stabilizers to prevent aggregation. However, they demand high energy input, can induce thermal or mechanical degradation, and sometimes yield broad size distributions [22,23]. Bottom-up approaches rely on controlled precipitation from supersaturated solutions, typically achieved through solvent displacement, antisolvent addition, or supercritical fluid technologies. Antisolvent precipitation is particularly attractive: by rapidly mixing a drug solution with a miscible nonsolvent, supersaturation is induced, nucleating fine particles whose size can be tuned by controlling mixing intensity, concentration, and stabilizer choice. Supercritical antisolvent (SAS) processes extend this concept by employing supercritical CO_2_, offering solvent removal advantages and tunable particle morphologies [24,25]. Hybrid methods combine bottom-up precipitation with downstream processes such as high-pressure homogenization or spray-drying to optimize stability and re-dispersibility [20,26]. Industrial translation favors continuous rather than batch processes, as continuous mixing devices such as confined impinging jets, static mixers, and coaxial jet mixers offer reproducibility, scalability, and high throughput. Continuous nanoprecipitation has been demonstrated to be able to produce lipid nanoparticles or polymer–drug systems with narrow size distributions and throughputs suitable for manufacturing [20,27]. At the patent level, numerous disclosures describe industrial reactors designed for nanoparticle production, emphasizing solvent handling, modular scalability, and integration with downstream purification. These developments underscore the industrial drive towards methods that deliver consistent particle sizes, low residual solvent, and GMP-compliant operations [28,29,30].

Antisolvent methods (classical and supercritical) operate by generating high supersaturation that triggers rapid nucleation while limiting uncontrolled growth. In classical solvent/antisolvent precipitation, one mixes an API solution (good solvent) with a large excess of antisolvent (poor solvent) so that supersaturation is instantaneous; control tools include shear (stirring, ultrasound), mixer geometry, microfluidic focusing, or turbulent jet mixing. Supercritical antisolvent (SAS) introduces supercritical CO_2_ as the antisolvent; CO_2_ extracts solvent, induces supersaturation and produces fine, often amorphous particles with low residual solvent [31]. A distinct and increasingly important implementation strategy is to deliver the two (or more) streams via a coaxial nozzle or coaxial jet: the core stream carries the API solution and the annular or surrounding stream carries antisolvent (or scCO_2_) so that mixing, shear, and diffusion occur in a highly controlled shear layer. Coaxial arrangements can be configured for laminar focusing (microfluidic coaxial flows) or for turbulent entrainment (coaxial turbulent jet mixers); each regime gives different mixing timescales and hence different nucleation/growth kinetics. Recent work demonstrates that continuous coaxial jet mixers can produce nanoparticles and lipid vesicles with narrow distributions and industrially relevant throughput [32,33,34]. Moreover, SAS with coaxial feed nozzles has been applied to produce fine particles of model lipophilic compounds: for instance, coaxial-nozzle SAS precipitation of drug/polymer systems yielded controlled particle morphology and reduced agglomeration [35]. Patents and recent patent applications also disclose systems combining coaxial feed geometries, high-shear mixing zones and downstream solvent extraction to produce pharmaceutical nanoparticles [36,37]. Therefore, coaxial jet antisolvent processing is a maturing route: it offers rapid mixing, controllable residence time distribution, and compatibility with continuous manufacturing. Remaining challenges include achieving reproducible sub-200 nm sizing for diverse lipophilic APIs, minimizing residual solvent to acceptable regulatory limits, achieving high encapsulation/loading when polymers or excipients are co-precipitated, and ensuring robust scale-out—all issues that require systematic parametric studies and process modeling [38,39]. These challenges highlight the need for process configurations that combine controllable hydrodynamics with continuous operation, enabling both reproducibility and scalability.

In this paper, we proposed addressing these gaps by developing a robust, scalable method to produce nanoliposomes and nanoparticles of lipophilic APIs, using curcumin as a model compound, via an anti-solvent coaxial jet system. Specifically, we design and build a coaxial nozzle apparatus in which a solution of curcumin (for nanoparticle formation) or phospholipids (for liposome formation) in ethanol is injected through an inner core flow, surrounded by a coaxial anti-solvent stream of water, with precise control of flow rates. We performed extensive characterization of the products in terms of particle size (typically in the 100–300 nm range) and polydispersity index (PDI < 0.2). We also proposed and validated a mathematical model of nucleation, growth and precipitation in the coaxial jet anti-solvent environment, relating key process parameters (flow ratios, mixing profile, supersaturation) to particle size and yield. Finally, we tested the model against experimental data for curcumin and liposomes, aiming to show that the method can produce nanoparticles (<200 nm, PDI < 0.2) with high encapsulation efficiency, reproducibility, and industrially relevant throughput, thereby offering a practical route to improving the bioavailability of curcumin and similar lipophilic APIs. By combining experiments, apparatus engineering and predictive modeling, this work bridges laboratory nanoprecipitation and practical manufacturing, aligning with recent continuous nanoparticle production developments and patented mixing systems. The outcomes are expected to provide not only a proof-of-concept for the proposed apparatus but also generalizable design principles that support broader application of coaxial antisolvent jet technology in the pharmaceutical industry.

## 2. Materials and Methods

### 2.1. Materials

Deionized water was used to prepare all the aqueous solutions. Ethanol absolute (CAS (64-17-5) was bought from Carlo Erba Reagents (Cornaredo, Italy). HCl 37% (CAS 7647-01-0) was provided by VWR International Srl (Milan, Italy), NaOH (CAS 1310-73-2), phenolphthalein (CAS 77-09-8), boric acid (H_3_BO_3_, CAS 10043-35-3), potassium iodate (KIO_3_, CAS 7758-05-06), potassium iodide (KI, CAS 7681-11-0) and sulfuric acid (H_2_SO_4_, CAS 7664-93-9) were bought from Merck (Rome, Italy).

Curcumin (CUR) (CAS 458-37-7) was used as a hydrophobic model compound and bought from Merck (Italy).

Curcumin powder was dissolved in absolute ethanol (≥99.8%) to prepare stock solutions at concentrations typically in the range of 1–3.5 g/L, depending on the specific experiment.

For nanoliposomes, a phosphatidylcholine-enriched solution was extracted from soy lecithin (PWD E322 (CAS 8002-43-5)) purchased from A.C.E.F. S.r.L. (Piacenza, Italy), following ethanol extraction and centrifugation, as described in [40].

All chemicals were of analytical grade and used as received.

### 2.2. Methods

#### 2.2.1. Coaxial Jet Mixer

Nanoparticles and nanoliposomes were produced using a coaxial jet mixer consisting of a stainless-steel inner needle (typically 23G or 25G) concentrically aligned within an outer tube. The inner channel delivered the solvent stream (ethanol containing dissolved lipid or curcumin), while the outer channel conveyed the antisolvent (water). Two independent precision dosing systems ensured stable and reproducible flow delivery [41]. The use of independent dosing systems ensures stable and reproducible flow conditions, which are essential for process scalability and continuous operation. Flow rates were adjusted to generate laminar, transitional, or turbulent hydrodynamic regimes, while maintaining or varying the Flow Velocity Ratio and Reynolds number as required.

The coaxial configuration was operated in vertical orientation, as this configuration minimized buoyancy-induced instabilities and promoted more uniform flow development, in agreement with our earlier investigations and preliminary testing [32].

#### 2.2.2. Particle Size Analysis

Hydrodynamic diameter and polydispersity index (PDI) were measured by dynamic light scattering using a benchtop Zetasizer Pro Red Label (Malvern Panalytical, Malvern, UK). Samples were diluted in deionized water to minimize multiple scattering and were equilibrated at 25 °C before measurement. Each reported value corresponds to the mean of at least three independent measurements (*n* ≥ 3), and results are expressed as mean ± standard deviation.

#### 2.2.3. Curcumin Quantification

Quantification of curcumin concentration and, when required, reaction products in micromixing experiments was performed using a UV–visible spectrophotometer operating between 200 and 600 nm. For curcumin, absorbance was evaluated at approximately 426 nm and calibration curves were prepared from ethanolic standards.

### 2.3. Experimental Workflow

The experimental campaign consisted of three interconnected steps: (i) fluid-dynamic characterization, (ii) nanoparticle and liposome production, and (iii) integration of modeling and process interpretation. Although reported sequentially, these activities were strongly interdependent.

#### 2.3.1. Fluid-Dynamic Analysis

Initial tests involved water–water systems.

Mixing performance was assessed by means of a fast acid–base neutralization reaction employed as a visual tracer of micromixing. Separate aqueous solutions of HCl (0.1 M) and NaOH (0.1 M) were injected through the outer tube and the inner needle of the coaxial mixer, respectively. Phenolphthalein was added to the NaOH stream, enabling direct visualization of the neutralization front through color change upon contact with the acidic stream. High-speed video acquisition combined with image analysis was used to measure the mixing length and to estimate characteristic mixing times. The mixing length $L_{\mathrm{mix}}$ was determined from image analysis as the axial distance from the needle exit at which the color intensity reached 90% of its fully mixed value. The characteristic mixing time was then estimated as $\tau_{\mathrm{mix}}=L_{\mathrm{mix}}/u_{\mathrm{avg}}$, where $u_{\mathrm{avg}}$ is the average flow velocity calculated from the total flow rate and the cross-sectional area of the outer tube.

In parallel, micromixing was quantified using the Villermaux–Dushman reaction, a competitive reaction system based on the competition between a quasi-instantaneous neutralization and a slower iodide–iodate reaction. Owing to its sensitivity to local concentration inhomogeneities at the molecular scale, this method allows micromixing to be assessed through the segregation index derived from the reaction yield [42,43]. Reagent concentrations, reported in Table 1, were selected following a previously validated protocol, in order to ensure that the characteristic reaction times of the iodide–iodate system were comparable to the expected micromixing timescale, thus allowing the segregation index to be sensitive to micromixing effects. I_3_^−^ concentration was quantified by UV–Vis spectrophotometry at 353 nm according to the Beer–Lambert law. The molar extinction coefficient was determined by calibration using iodine/iodide solutions.

Micromixing intensity was quantified through the segregation index $X_{s}$, defined as the ratio between the experimentally determined reaction yield Y and the yield under total segregation conditions $Y_{ST}$:

(1) $$X_{s}=\frac{Y}{Y_{ST}}$$

where Y represents the fraction of injected protons involved in the Dushman reaction, as determined from the measured triiodide concentration, and $Y_{ST}$ corresponds to the theoretical yield in the case of complete segregation. By definition, $X_{s}=0$ indicates ideal micromixing, while $X_{s}=1$ corresponds to total segregation [44].

#### 2.3.2. Nanoparticle Production

Curcumin nanoparticles were produced by injecting ethanolic curcumin solution through the inner needle and water or water + PVP solution through the outer channel. Operating conditions were selected to evaluate both laminar and turbulent regimes while maintaining a defined water/ethanol ratio. Samples were collected continuously, characterized by DLS and spectrophotometry, and, when necessary, subjected to tangential-flow filtration before quantification [45].

#### 2.3.3. Liposome Production

For liposomes, phospholipids (predominantly phosphatidylcholine) dissolved in ethanol served as the inner stream, while water acted as an antisolvent. Flow rate and phosphatidylcholine concentration were varied systematically, and the resulting suspensions were evaluated in terms of size and size distribution, whereas encapsulation behavior and stability were not subjects of this analysis, having been studied elsewhere [43].

## 3. Modeling

The precipitation of curcumin nanoparticles in the coaxial jet mixer (CJM) was described using a coupled computational fluid dynamics (CFD)–population balance equations (PBE) framework, implemented in COMSOL Multiphysics^®^ 6.1 and solved at steady state. The fluid dynamics of the coaxial mixing section were described by the incompressible Navier–Stokes equations, closed with a two-equation eddy-viscosity turbulence model, while nanoparticle formation and growth were described by a one-dimensional population balance equation (PBE) in particle size, solved by the method of moments. The model was parametrized using independently measured or literature values of fluid properties and species solubility and growth kinetics, and it was validated against the experimental data reported in this work [46]. The model provides a framework that can be extended to scale-up analysis by preserving dimensionless operating parameters.

The following set of equations describes the coupled evolution of flow, species transport, and particle population. For clarity, the main physical meaning of each equation is briefly summarized before introducing the full mathematical formulation.

### 3.1. Geometry and Operating Conditions

The computational domain reproduced the experimental CJM geometry used in the dynamic experiments. The mixer consists of a straight cylindrical outer tube, into which a 23G stainless-steel needle delivers the organic phase (ethanol containing dissolved curcumin, and, when present, PVP). The aqueous antisolvent flows in the annular region between the needle and the outer tube.

The outer tube had an internal diameter of 3.0 mm and an overall length of 20.5 cm, corresponding to the length effectively used in the experiments. The 23G needle had an internal diameter of 337 μm and an external diameter of 641.4 μm. These dimensions, together with the experimental flow rates of inner and outer streams, define the Reynolds numbers of the two inlets and the residence time distribution along the mixing section.

**Figure 1:**
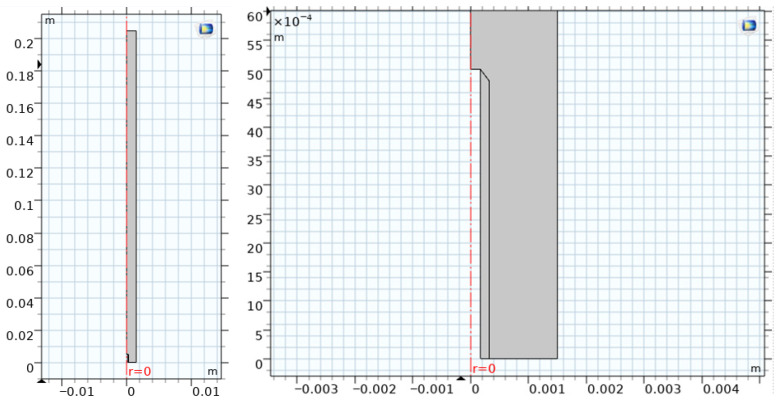
Computational domain adopted for CFD simulations. The system was modeled as axisymmetric, including the needle exit region and the downstream mixing zone. The internal flow inside the needle was not simulated; instead, a prescribed velocity boundary condition was imposed at the needle exit. The domain includes the needle-exit region and the downstream mixing zone. (**Left**): full computational domain. (**Right**): zoomed view of the needle-exit region.

Exploiting the cylindrical symmetry of the system, the simulations were carried out in a two-dimensional axisymmetric domain. A schematic representation of the computational domain, including the two inlets (needle and annulus), the outlet boundary, and the symmetry axis, is reported in Figure 1. The mesh was refined in the vicinity of the needle tip and in the shear layers developing downstream, to resolve velocity and concentration gradients in the mixing region. The main geometric parameters and their nominal values are summarized in Table 2.

The simulations were run at the same flow-rate combinations adopted in the experiments, spanning one laminar, one transition and one turbulent flow regimes. The corresponding inlet velocities were used here as boundary conditions for the hydrodynamic model.

### 3.2. Hydrodynamics and Turbulence Modeling

The flow field in the coaxial mixer was described by the incompressible Navier–Stokes and continuity equations in axisymmetric coordinates. In vector form, the continuity and momentum equations read [47]:

(2) ∇⋅ρu=0

(3) $$\nabla p+\rho \left(\nabla \cdot uu\right)=-\nabla \cdot \left(\tau^{\left(v\right)}+\tau^{\left(t\right)}\right)+\rho g$$

where u is the velocity vector, p is the pressure, ρ is the fluid density (of the mixture ethanol–water), $\tau^{\left(v\right)}$ is the viscous stress tensor, and $\tau^{\left(t\right)}$ is the turbulent stress tensor.

In the turbulent cases, the Navier–Stokes equations were solved in Reynolds-averaged form (RANS), following the standard decomposition of the instantaneous velocity and pressure fields into mean and fluctuating components [48,49]. The Reynolds stress tensor emerging from the averaging procedure was closed by invoking the Boussinesq eddy-viscosity hypothesis:

(4) $$\tau_{i,j}^{\left(t\right)}=\rho \bar{u_{i}^{\prime }u_{j}^{\prime }}=2\mu_{t}S_{ij}\frac{2}{3}\rho k\delta_{ij}$$

where $\bar{u_{i}^{\prime }u_{j}^{\prime }}$ are the Reynolds stresses, $S_{ij}=\frac{1}{2}\left(\partial \bar{u_{i}}/\partial x_{j}+\partial \bar{u_{j}}/\partial x_{i}\right)$ is the mean rate-of-strain tensor, k is the turbulent kinetic energy, and $\mu_{t}$ is the turbulent (eddy) viscosity.

The eddy viscosity was related to the turbulence scales through the classical k–ε model [50]:

(5) $$\mu_{t}=\rho C_{\mu }\frac{k^{2}}{\varepsilon }$$

where ε is the turbulent dissipation rate and $C_{\mu }$ is a model constant. The transport equations for k and ε were written as

(6) $$\rho u\cdot \nabla k=\nabla \cdot \left[\left(\mu +\frac{\mu_{t}}{\sigma_{k}}\right)\nabla k\right]+P_{k}-\rho \varepsilon$$

(7) $$\rho u\cdot \nabla \varepsilon =\nabla \cdot \left[\left(\mu +\frac{\mu_{t}}{\sigma_{\varepsilon }}\right)\nabla \varepsilon \right]+C_{1\varepsilon }\frac{\varepsilon }{k}P_{k}-C_{2\varepsilon }\rho \frac{\varepsilon^{2}}{k}$$

with $G_{k}$ the production rate of turbulent kinetic energy due to mean velocity gradients, and $\sigma_{k}=1.0$, $\sigma_{\varepsilon }=1.3$, $C_{1\varepsilon }=1.44$, $C_{2\varepsilon }=1.92$ and $C_{\mu }=0.09$ the usual model constants [49,50].

(8) $$P_{k}=\mu_{t}\left(\nabla u:\left(\nabla u+\nabla u^{T}\right)-\frac{2}{3}{\left(\nabla \cdot u\right)}^{2}\right)-2\rho k\frac{\varepsilon^{2}}{k}$$

No-slip boundary conditions were imposed on the solid walls. Fully developed velocity profiles were prescribed at the two inlets, computed from the specified volumetric flow rates and, for the turbulent cases, from standard inlet turbulence intensity and length-scale estimates [48]. At the outlet, a constant pressure boundary condition was applied.

### 3.3. Ethanol–Water Mixing and Property Correlations

The nanoprecipitation process is controlled by local supersaturation of curcumin following mixing of ethanol feed with the aqueous antisolvent. The local composition of the solvent mixture was described by the transport of the ethanol mass fraction $\omega_{e}$:

(9) $$\rho u\cdot \nabla \omega_{e}=\nabla \cdot \left[\rho \left(D_{m}+D_{t}\right)\cdot \nabla \omega_{e}\right]$$

where $D_{m}$ is the molecular diffusivity of ethanol in water and $D_{t}$ is the turbulent diffusivity, modeled as

(10) $$D_{t}=\frac{\mu_{t}}{\rho N_{Sc}^{t}}$$

where $N_{Sc}^{t}$ is the turbulent Schmidt number, assumed constant and equal to 0.71 [48].

The density $\rho \left(\omega_{e}\right)$ and dynamic viscosity $\mu \left(\omega_{e}\right)$ of the ethanol–water mixture were correlated against literature data for binary water–ethanol systems at 25 °C, using polynomial fits to the experimental measurements reported by [51,52]. Molecular diffusivities were obtained from the data of Klinov and Anashkin [53] and interpolated as a function of composition to provide smooth $D_{m}\left(\omega_{e}\right)$ correlations.

**Figure 2:**
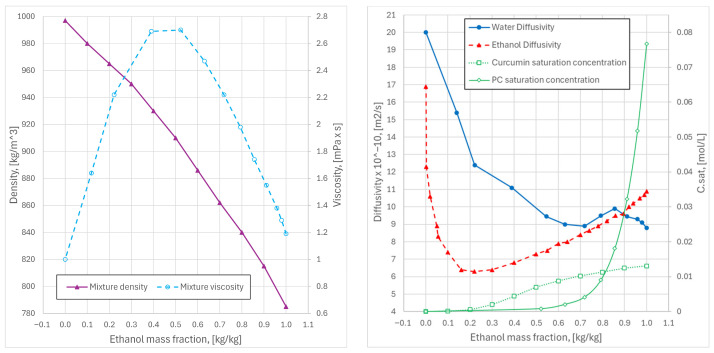
On the left, density (**left axis**) and viscosity (**right axis**) of water–ethanol mixtures; on the right, diffusivities of water and ethanol (**left axis**), and saturation concentration of curcumin and of phosphatidylcholine (PC) in water–ethanol solution (**right axis**). All the data plotted versus the ethanol mass fraction $\omega_{e}$.

The saturation solubility of curcumin in the mixed solvent was parameterized by regressing literature data for its solubility in ethanol and in aqueous ethanol mixtures at 25 °C. Direct solubility data for phosphatidylcholine (PC) in ethanol–water mixtures are scarcely reported in the literature. Following similar works [54], the solubility curve reported for cholesterol [54] was adopted as a reference for PC solubility, providing a practical basis for modeling. All the data are summarized in Figure 2.

The supersaturation ratio

(11) $$S=\frac{C_{s}}{C_{s,\mathrm{sat}}\left(\omega_{e}\right)}$$

could be evaluated locally, where $C_{s}$ is the total solute concentration (dissolved plus in the particulate phase) and $C_{s,\mathrm{sat}}\left(\omega_{e}\right)$ is the solute saturation solubility. The overall strategy is consistent with standard descriptions of solution crystallization and antisolvent precipitation [55,56].

### 3.4. Population Balance Model and Dissolved Solute Transport Equation

Nanoparticle formation and growth were described with a population balance equation (PBE) in terms of the particle size coordinate L (characteristic diameter or equivalent length). The population balance equation (PBE) accounts for the evolution of the particle size distribution as a result of nucleation and growth processes. At each spatial location in the mixer, the number density function $n\left(L,x\right)$ satisfies the following equation at steady state [55,57]:

(12) $$\nabla \cdot un\left(L;x\right)=\nabla {\;\cdot \;D}_{T}\nabla n\left(L;x\right)+J(L,S)+\frac{\partial }{\partial L}\left[G(S)\;n\left(L;x\right)\right]$$

where J(L,S) is the nucleation rate and G(S) is the size-independent growth rate (assumed to depend only on local supersaturation).

In order to make the coupling with CFD tractable, the PBE was solved using the method of moments (MOMs). The j-th moment of the size distribution is defined as

(13) $$m_{j}\left(x\right)=\int_{0}^{\infty }L^{j}n\left(L,x\right),dL$$

By multiplying Equation (12) by $L^{j}$ and integrating over size space, a hierarchy of transport equations for the moments $m_{j}$ is obtained [55,57]. The PBE solution obtained by MOMs and used here is

(14) $$\left.(u\nabla <m_{j}>\right)=\left(\nabla \cdot \left(D^{\left(t\right)}\nabla <m_{j}>\right)\right)+h_{j},\;j=0,..,7$$

In Equation (12), the source terms are given by

(15) $$h_{j}=JL_{c}^{j}+\left\{\begin{array}{cc} 0 & j=0\\ jG<m_{j-1}> & j>0 \end{array}\right.$$

For the present system, a pure nucleation–growth mechanism without aggregation was adopted, so that closed-form expressions for the moment source terms could be written in terms of the local supersaturation S.

Nucleation rate (expressed in nuclei/(m^3^s)) was described by:

(16) $$J\left(L\right)=\left\{\begin{array}{c} J_{c}\left(S\right)\;\;\;\;\;\;\;\;\;\;L=L_{c}\\ 0\;\;\;\;\;\;\;\;\;\;\;\;\;\;\;\;\;L\neq L_{c} \end{array}\right.,\;\mathrm{with}\;J_{c}(S)=Aexp\left(\frac{-b}{{\left(lnS\right)}^{2}}\right)$$

where A is the pre-exponential factor and b is a thermodynamic parameter related to interfacial energy. In this work, b was obtained from literature, while A was treated as a fitting parameter against experimental particle size distributions. $L_{c}=1\;nm$ and $L_{c}=10\;nm$ represent the nuclei size of curcumin nanoparticles and liposomes, respectively. This choice reflects the different characteristic length scales associated with molecular aggregation and vesicle self-assembly. For curcumin nanoparticles, nucleation corresponds to the formation of molecular clusters stabilized by intermolecular interactions. In contrast, liposome formation involves the self-assembly of lipid bilayers into closed vesicular structures, which requires a minimum number of lipid molecules to sustain bilayer curvature and vesicle closure. As a result, a larger nucleus size was adopted.

The crystal growth rate (expressed in nm/s) was modeled by adapting the mechanistic approach proposed by Shin, Devos [58] and Iggland and Mazzotti [59], where the growth driving force is typically expressed as:

(17) $$G=k_{g}C_{s,sat}\left(\omega_{e}\right)\left(S-S^{*}\right)$$

where S* represents the equilibrium saturation ratio based on the Gibbs–Thomson equation, which accounts for size-dependent solubility. In this study, given the high supersaturation levels typical of turbulent jet mixing, the capillary effect was assumed to be negligible compared to the primary precipitation driving force. Consequently, $S^{*}$ was set to 1. The growth rate constant, $k_{g}$, was determined by fitting the model to experimental data. In this framework, nucleation determines the formation rate of new particles, while growth controls their size increase, both processes being driven by the local supersaturation field resulting from mixing.

In the MOM formulation implemented here, a finite number of low-order moments (j = 0,..,7) were transported in the CFD domain, and the experimentally accessible quantities such as total particle volume concentration and volume-weighted mean diameter were recovered from the computed moments at the mixer outlet. In particular, the intensity-weighted mean diameter $d_{76}$ was computed from the particle size moments according to $d_{76}=m_{7}/m_{6}$, where $m_{j}$ denotes the j-th moment of the particle size distribution. This definition is consistent with an intensity-weighted average, as higher-order moments emphasize the contribution of larger particles. The choice of $d_{76}$ allows a direct comparison with the Z-average diameter obtained from dynamic light scattering (DLS), which is also strongly weighted toward the larger size fraction of the distribution.

In addition to the particulate phase, the solute was also transported in the dissolved phase, with concentration $C_{s}$. As crystal nucleation and growth remove mass from the liquid phase, the solute concentration decreases accordingly. The steady-state solute transport equation can therefore be written as:

(18) $$u\nabla C_{s}=\left(\nabla \cdot \left(D_{eff}\nabla C_{s}\right)\right)-\frac{h_{3}\rho_{s}}{M_{s}}$$

where $h_{3}=JL_{c}^{3}+3G<m_{2}>$ is the source term for the second moment $m_{2}$, $\rho_{s}$ is the solute crystal density and $M_{s}$ is the solute molar mass (the density is $1390\;[kg/m^{3}]$ for both curcumin and phosphatidylcholine, and the molar mass is 0.368  [kg/mol] for curcumin and 0.750 [kg/mol] for phosphatidylcholine).

### 3.5. Numerical Solution and Coupling Strategy

The hydrodynamics, scalar mixing and population balance equations were solved in a sequentially coupled manner. First, the velocity and pressure fields, along with the solvent composition, were obtained by solving the RANS equations with the k–ε closure coupled with the ethanol mass fraction equation until convergence. Then, the PBE moments and the solute transport equation were integrated using the converged flow and composition fields, following an approach similar to other CFD–PBE studies of reactive precipitation and co-precipitation processes [60].

The finite-element mesh was progressively refined until the relative variation in the outlet-averaged third moment (m_3_) and volume-weighted mean diameter (d_43_) between two consecutive mesh refinements was below 1%. Solver tolerances were set to ensure residuals lower than 10^−6^ for all transported variables. The resulting CFD–PBE model was then used to rationalize and semi-quantitatively predict the influence of flow regime (laminar vs. turbulent), flow-rate ratio, and solvent composition on the experimentally observed nanoparticle size distributions.

## 4. Results and Discussion

### 4.1. Fluid-Dynamic Characterization

The coaxial jet configuration determines how rapidly solvent and antisolvent interact, how steep composition gradients develop, and ultimately the environment in which precipitation or vesicle self-assembly takes place. For this reason, the hydrodynamics of the mixer were first examined systematically, combining tracer experiments, neutralization tests, and quantitative image analysis.

#### 4.1.1. Flow Visualization and Regime Identification

**Figure 3:**
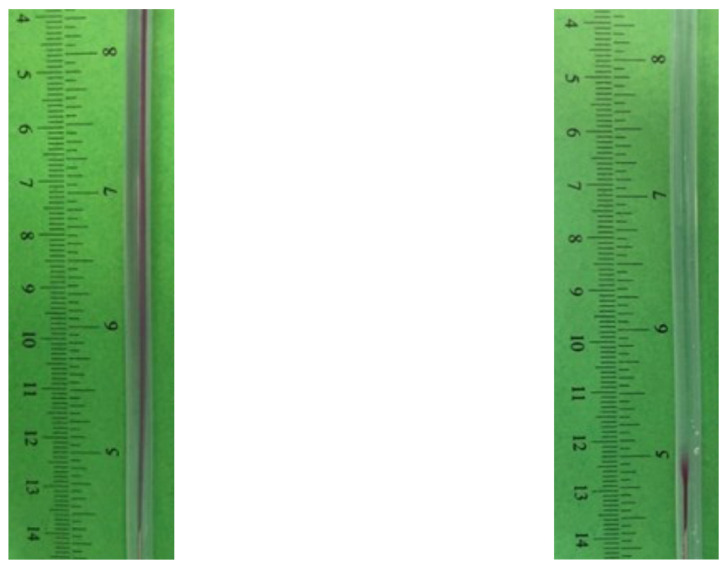
Representative frames from video analysis of the acid–base neutralization system (HCl–NaOH with phenolphthalein) in laminar and turbulent regimes. In the laminar regime (**left**), the persistence of the purple phenolphthalein color indicates incomplete neutralization and poor mixing along the observation length. In contrast, in the turbulent regime (**right**), the color rapidly disappears after a short entrance region, indicating fast neutralization and effective mixing. The axial distance from the needle exit at which complete discoloration occurs was used to determine the mixing length *L*_mix_, subsequently employed for the estimation of the mixing time.

The apparatus consists of a stainless-steel 23G needle delivering the organic stream concentrically into a cylindrical tube carrying the aqueous phase. Preliminary tests confirmed that the vertical configuration avoids buoyancy-driven segregation of the lighter ethanolic jet, which is instead clearly visible in horizontal operation, in line with previous observations on coaxial systems [32,33]. Under vertical operation, the injected jet remains centered and symmetric across the range of flow rates investigated; see Figure 3.

Visualization was performed by adding phenolphthalein to the inner stream and monitoring neutralization when it contacted the outer acidic solution. Depending on operating conditions, three qualitatively distinct flow behaviors could be recognized:

- Laminar focusing—the inner jet retains a coherent core, with limited entrainment and incomplete neutralization along the axial direction.
- Transitional flow—intermittent perturbations appear, producing local deformation of the jet and irregular interfaces.
- Jet-like/turbulent conditions—the colored core rapidly disappears after the needle outlet, indicating intense deformation and fast dilution of the injected stream.

**Figure 4:**
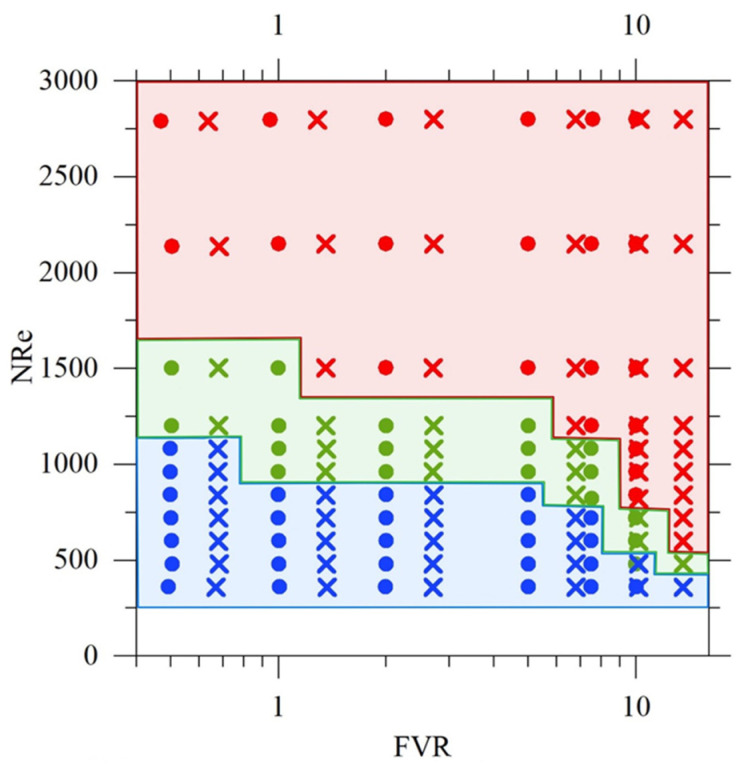
Hydrodynamic regime map (laminar—blue, transitional—green, turbulent—red) as a function of the Reynolds number and flow velocity ratio (FVR), obtained from image analysis of the acid–base neutralization system (HCl–NaOH with phenolphthalein) in a coaxial injection mixer operating under water–water conditions. Filled circles correspond to experiments performed with the 23G needle, while crosses indicate experiments carried out with the 25G needle. The different flow regimes were identified based on qualitative changes in the mixing pattern observed in the images.

These behaviors were mapped as a function of Reynolds number $N_{Re}$ and Flow Velocity Ratio FVR, as shown in Figure 4, confirming that regime boundaries depend on both overall inertia and relative momentum between the two streams, consistently with previously reported coaxial jet studies [33,61].

#### 4.1.2. Characteristic Mixing Lengths and Times

For each operating point, the axial distance at which the tracer became visually indistinguishable was measured (mixing length, $L_{mix}$). Dividing $L_{mix}$ by the mean axial velocity yielded an operational characteristic time, $\tau_{mix}$, providing a simple descriptor of how rapidly composition gradients decay under the imposed conditions.

**Figure 5:**
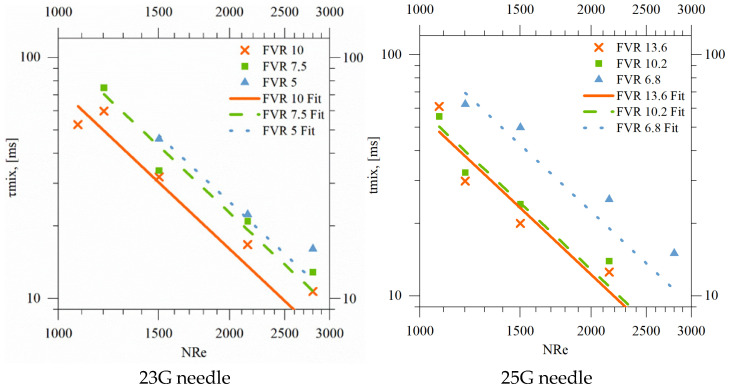
Characteristic mixing time $\tau_{mix}$ as a function of Reynolds number for different FVR values and for two different needles. Symbols: experiments; lines: power-law fits.

A clear trend emerged: $\tau_{mix}$ decreased monotonically with increasing $N_{Re}$ and FVR, as reported in Figure 5. The dependence could be described satisfactorily by a power-law expression of the form:

(19) $$\tau_{\mathrm{mix}}=\alpha \cdot {N_{Re}}^{-n}$$

with n≈2.22.

In line with previous observations for coaxial injection systems [32], the prefactor α weakly exhibited a systematic dependence on the flow velocity ratio, which could be approximated by a linear relationship. Similar functional relationships have been reported for confined and coaxial jets, where increasing inertia accelerates jet breakup and axial dilution [32,33,62]. In our case, the correlation allows a practical identification of operating windows with short characteristic times, without assuming a priori which physical mechanism (shear-induced stretching, entrainment, or small-scale mixing) dominates.

It is worth stressing that $\tau_{mix}$, as used here, is an operational metric, not a direct measurement of micro-scale mixing. It reflects the combined effects of jet breakup, entrainment, convective transport and diffusion, and is therefore better interpreted as a global indicator of how fast the injected phase loses its identity in the main stream.

The Villermaux–Dushman reaction was employed to independently assess micromixing through the segregation index $X_{s}$ for selected operating conditions using the 23G needle. Table 3 summarizes the corresponding flow velocity ratio, Reynolds number, flow regime, mixing time $\tau_{\mathrm{mix}}$ obtained from the acid–base visualization experiments, and the measured $X_{s}$ values.

As expected, in the turbulent regime $X_{s}$ approached zero, indicating efficient micromixing and dominant consumption of the common reactant by the quasi-instantaneous reaction. Higher $X_{s}$ values were instead observed under laminar and transitional conditions, reflecting incomplete micromixing.

**Figure 6:**
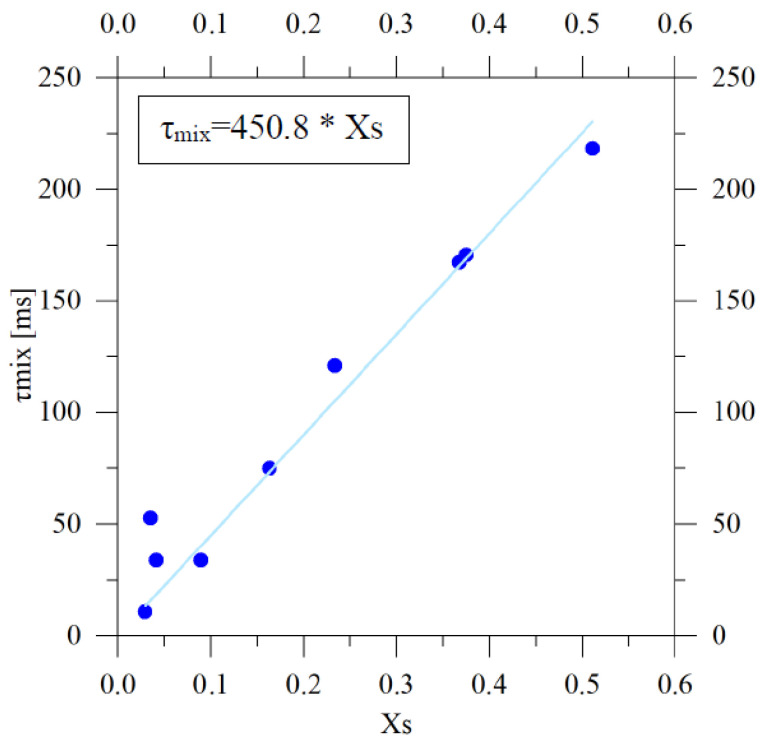
Correlation between the segregation index $X_{s}$ and the characteristic mixing time $\tau_{\mathrm{mix}}$, showing an approximately linear relationship ($\tau_{\mathrm{mix}}=450.8\;X_{s}$) for the Villermaux–Dushman and acid–base visualization experiments performed with the 23G needle.

Figure 6 shows the relationship between the segregation index and the characteristic mixing time, revealing an approximately linear correlation between $X_{s}$ and $\tau_{\mathrm{mix}}$. The fact that two independent experimental approaches—visual mixing-length analysis and spectrophotometric determination of $X_{s}$—lead to consistent trends provides strong evidence for the robustness of the proposed characterization and confirms the reliability of $\tau_{\mathrm{mix}}$ as an operational indicator of mixing efficiency. An additional advantage of the Villermaux–Dushman method is that micromixing characterization can be performed by spectrophotometric analysis alone, without the need for visual access to the mixing zone, making the approach applicable to opaque or geometrically constrained systems.

### 4.2. Nanoparticles Production and Characterization

The hydrodynamic analysis shows that the coaxial mixer spans a wide spectrum of flow conditions, from laminar focusing to strongly entrained jet-like regimes. This evolution has direct consequences for the environment experienced by solutes or lipids when they are brought into contact with the antisolvent, thereby influencing nucleation, assembly, and subsequent aggregation processes.

In this section, we report the behavior of two representative systems tested in the mixer: lipid vesicles (nanoliposomes) and curcumin nanoparticles. These systems were selected because they exhibit markedly different solubility and aggregation behaviors, allowing a broader interpretation of how the characteristic mixing time influences the final product.

The fluid-dynamic operating conditions reported in Table 4 were intentionally selected to be identical for both nanoparticle systems investigated (liposomes and curcumin nanoparticles). For each experiment, the same inner and outer flow rates were employed, resulting in identical flow velocity ratios (FVR) and, consequently, comparable characteristic mixing times $\tau_{\mathrm{mix}}$. Owing to the identical flow-rate ratios, the dilution experienced by the injected stream upon mixing with the antisolvent was the same in both systems, leading to the same nominal outlet concentrations. This choice allows the effects of hydrodynamic conditions—through $\tau_{\mathrm{mix}}$ and flow regime—to be isolated from compositional effects, enabling a meaningful comparison between lipid self-assembly and molecular nanoprecipitation processes. Differences observed between laminar and turbulent regimes, on the other hand, are significantly larger than the experimental variability, confirming the robustness of the identified trends.

#### 4.2.1. Nanoliposomes Formation

Liposomal suspensions were produced by injecting ethanolic lipid solutions into the aqueous stream at different flow rates (Table 4) and lipid concentrations ($C_{PC}\;=\;24,\;44,\;and\;94\;g/L$). The experiments clearly showed that moving from laminar to faster regimes leads to a systematic reduction in vesicle size, accompanied by narrower distributions and good encapsulation performance. Similar trends have been reported in continuous liposome manufacturing approaches based on nanoprecipitation and rapid dilution, where faster solvent displacement results in shorter assembly times and smaller liposomes (≈100–200 nm) with reduced polydispersity (PDI typically below 0.20) [62,63].

However, the present results indicate that this reduction should not be interpreted exclusively as evidence of micromixing-controlled assembly. Rather, the observed behavior can be rationalized by considering that faster entrainment reduces the persistence of composition gradients, resulting in more uniform supersaturation conditions around the assembling lipid aggregates. This observation is consistent with reports showing that both diffusion-driven and convection-enhanced mixing may contribute to liposome size control in continuous devices [64,65].

**Figure 7:**
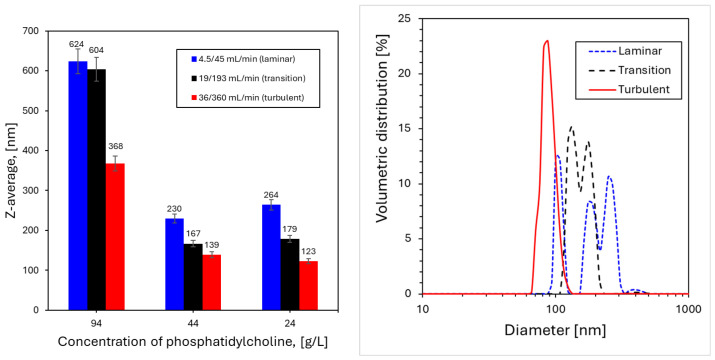
Size (on the (**left**)) and size distributions (on the (**right**)) of nanoliposomes produced at different phosphatidylcholine concentrations and under different fluid-dynamic regimes. Increasing lipid content results in larger and broader distributions, while higher flow rates reduce the vesicle size. Data are reported as mean ± standard deviation (n ≥ 3).

At fixed flow conditions, increasing lipid concentration led to larger vesicles and broader distributions, likely due to the increased probability of inter-vesicle collisions during the early stages of assembly; the experimental results are given in Figure 7. For example, at CPC = 44 g/L, the mean diameter decreased from approximately 230 ± 9.2 nm under laminar conditions to 139 ± 5.6 nm under turbulent conditions. Comparable trends have been described for both simil-microfluidic and turbulent nanoprecipitation systems, where concentration plays a role analogous to local supersaturation [63,64].

It should also be considered that increasing phosphatidylcholine concentration in the inner stream may alter not only the amount of lipid available for self-assembly, but also the rheological properties of the injected phase, particularly its viscosity. Such a change may influence the local momentum balance between the inner and outer streams, thereby modifying jet deformation, entrainment, and the effective flow velocity ratio in the near-field mixing region. From this perspective, the effect of phosphatidylcholine concentration on liposome size may reflect a genuine coupling between compositional and hydrodynamic factors, rather than a purely formulation-driven contribution.

Overall, the liposome results confirm that shorter operational mixing times favor smaller and more uniform structures, while lipid concentration emerges as an additional key parameter controlling vesicle growth. In this context, the effect of lipid concentration may arise not only from increased lipid availability, but also from possible changes in the effective viscosity of the injected phase, which could locally modify the flow regime and mixing efficiency. These findings indicate that liposome size is governed by the interplay between hydrodynamic mixing and lipid availability, rather than by a single dominant mechanism.

From a pharmaceutical perspective, encapsulation efficiency (EE) represents a key performance parameter for liposomal systems, as it directly determines the fraction of active compound effectively retained within the vesicles. Although a detailed quantitative analysis of EE is beyond the scope of the present work, and it cannot be discussed since no molecule has been loaded into the liposomes during these tests, the observed dependence of liposome size and formation conditions on mixing time provides important mechanistic insight.

In particular, under fast mixing conditions (short $\tau_{mix}$), the rapid and more homogeneous solvent exchange is expected to favor synchronized lipid self-assembly, reducing the probability of solute leakage during vesicle formation and promoting more efficient encapsulation. Conversely, under laminar or weakly mixed conditions, the persistence of composition gradients may lead to heterogeneous assembly environments, potentially resulting in less efficient or less reproducible encapsulation.

These considerations suggest that hydrodynamic control of mixing not only governs particle size but may also play a critical role in determining encapsulation performance, reinforcing the importance of process–formulation coupling in continuous nanocarrier production.

#### 4.2.2. Curcumin Nanoprecipitation

Curcumin nanoparticles were produced using the same mixer, enabling a comparison between lipid self-assembly and molecular precipitation phenomena. Curcumin presents very low aqueous solubility and rapidly forms supersaturated domains upon contact with water, thus providing a sensitive probe for mixing effects.

**Figure 8:**
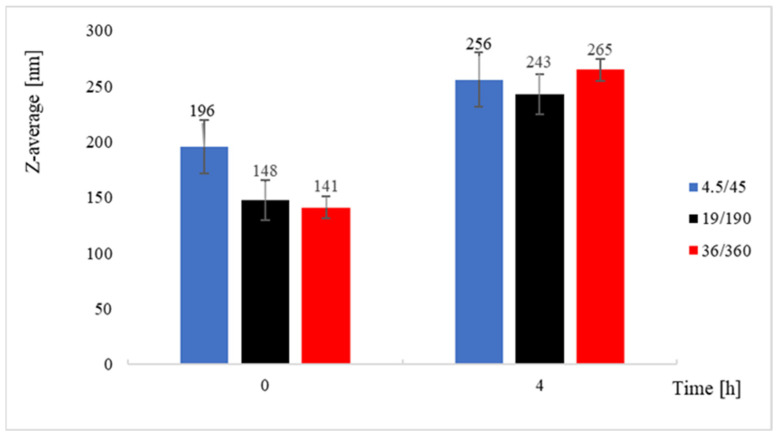
Z-average of curcumin nanoparticles produced in different fluid-dynamic conditions, as produced and after 4 h. As already noted, higher flow rates reduce the particle size. The increase with time is indicative of an aggregation process. Data are reported as mean ± standard deviation (n ≥ 3).

The experiments demonstrated that faster hydrodynamic regimes led to markedly smaller nanoparticles, as reported in Figure 8, in agreement with the general framework of nanoprecipitation, where shorter characteristics mixing times suppress growth and favor nucleation-dominated conditions. Such behavior has been extensively reported for flash-type precipitation systems, confined impinging jet mixers, and microfluidic devices [66,67].

Nonetheless, at prolonged storage times, curcumin suspensions displayed progressive aggregation and size increase, as shown in Figure 8. This result highlights that the beneficial effect of rapid dilution during particle formation may be partially counterbalanced by Ostwald ripening or particle–particle interactions in the absence of stabilizers. Similar instabilities have been reported in nanoprecipitated curcumin formulations when insufficient steric or electrostatic stabilization is present [66].

Although shorter mixing times clearly favor the formation of smaller particles, the results indicate that the final particle size and its temporal evolution cannot be explained by $\tau_{\mathrm{mix}}$ alone, pointing to the contribution of post-formation processes rather than to differences in the initial mixing stage.

#### 4.2.3. Comparative Interpretation

When considered together, the two systems show coherent and complementary behavior:

- Shorter operational mixing times correlate with smaller primary structures;
- Concentration governs the extent of aggregation during and after formation;
- Rapid formation does not automatically guarantee long-term stability.

The results therefore reinforce the conceptual view that the coaxial mixer primarily modifies the overall timescale over which solvent displacement and solute rearrangement occur. The operational mixing time $\tau_{\mathrm{mix}}$, while not being a pure micromixing timescale, integrates the effects of convective mixing, entrainment and turbulent micromixing. The implications of this observation are important for process scale-up, as it suggests that matching $\tau_{mix}$ may be more relevant than exactly reproducing local turbulent characteristics. These effects are also expected to influence encapsulation behavior, as more homogeneous mixing conditions may favor more uniform solute incorporation during particle or vesicle formation.

### 4.3. Modeling Results

The experimental work described in the previous sections highlights how the coaxial jet mixer allows control of the characteristic mixing time across a wide range of operating conditions. To rationalize the observed changes in nanoparticle characteristics, a coupled computational framework was developed integrating: (i) computational fluid dynamics (CFD), (ii) ethanol–water mixing, and (iii) population balance modeling (PBE) for nucleation and growth. This section summarizes the main outcomes of this integrated analysis and compares them with the experimental observations. The following results are thus presented to highlight how the model links hydrodynamic conditions, mixing time, and particle formation mechanisms.

#### 4.3.1. Hydrodynamics and Solvent Displacement Fields

**Figure 9:**
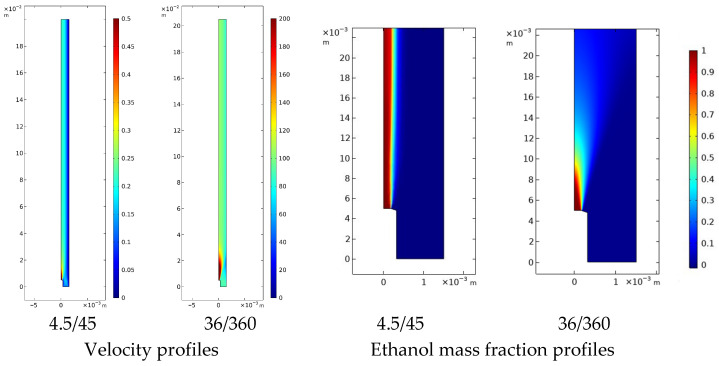
Simulated velocity and ethanol mass fraction profile for representative laminar (4.5/45) and turbulent (36/360) operating conditions. Rapid dilution of the ethanolic stream is observed at high total flow rates, while persistent composition gradients remain under laminar conditions.

The CFD simulations were performed assuming axisymmetric geometry and using a k–ε turbulence closure, consistent with common practice for pipe and jet configurations. The model quantitatively reproduced the progressive transition already qualitatively observed in tracer experiments: at low flow rates, the jet remained relatively focused, while at higher flow rates, strong entrainment promoted rapid dilution of the ethanolic core. The computed ethanol mass fraction fields demonstrated that, under turbulent operation, the inner stream loses its identity within a few millimeters downstream of the needle, whereas under laminar conditions, significant composition gradients persist along the axial direction. Both the velocity profiles and the ethanol mass fraction profiles are reported in Figure 9. This transition is accompanied by a marked decrease in the characteristic mixing length, in agreement with the experimental estimates derived from tracer fading.

Such behavior is in line with reports from coaxial and impinging mixers, where faster entrainment reduces concentration gradients and shortens the effective time available for particle growth [33,61].

#### 4.3.2. Curcumin Nanoparticle and Liposome Formation

**Figure 10:**
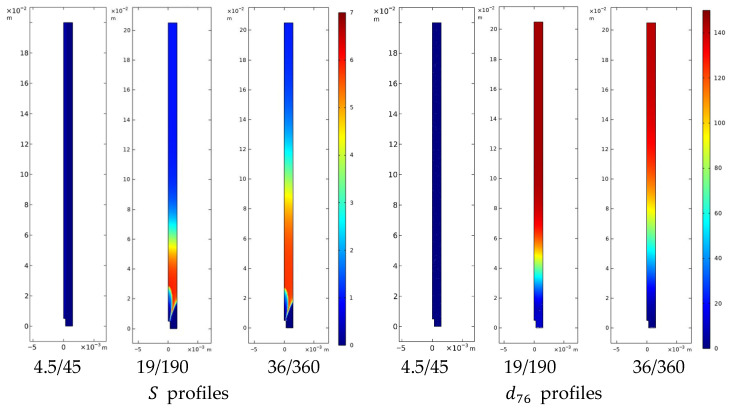
Computed spatial profiles for curcumin nanoparticles across different flow conditions. The contours illustrate the transition of the supersaturation ratio (S) and the intensity-weighted mean diameter ($d_{76}$) from laminar focusing (4.5/45 mL/min) to fully turbulent conditions (19/190 and 36/360 mL/min).

**Figure 11:**
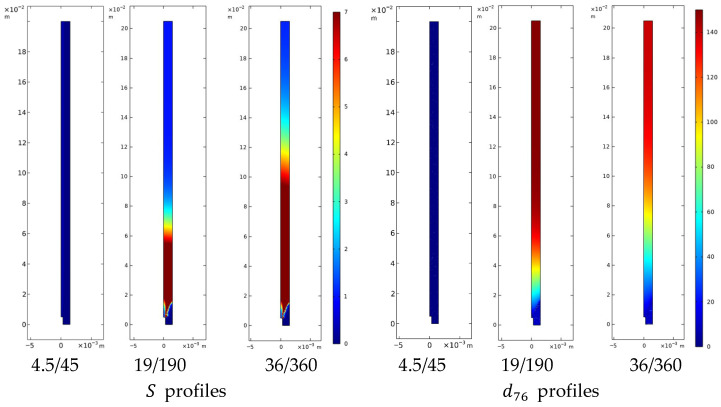
Computed spatial profiles for liposomes under different flow conditions. The contours illustrate the spatial distribution of the supersaturation ratio (S) and the intensity-weighted mean diameter ($d_{76}$) as the flow regime shifts from laminar focusing (4.5/45 mL/min) to increasingly turbulent conditions (19/190 and 36/360 mL/min).

To interpret nanoparticle formation, a PBE framework was adopted, including nucleation and growth contributions, coupled to the local supersaturation computed from the ethanol–water field and solubility data. The simulation results, shown in Figure 10 for curcumin and in Figure 11 for liposomes, reveal consistent trends across both systems, highlighting the dominant role of mixing intensity on particle final attributes. The simulated trends are consistent with the experimental observations, with deviations discussed in Section 4.3.2.

The model allows for a comparison across three regimes. First, under laminar focusing, the simulations predict negligible supersaturation and particle formation within the computational domain. In this idealized strictly coaxial configuration, the organic and aqueous streams flow with minimal interfacial contact. However, experimental observations of particles in these conditions suggest that molecular diffusion and unavoidable minor mechanical misalignments trigger a slow mixing process, leading to localized nucleation and extended growth times, resulting in increased particle size.

Second, under turbulent conditions, both systems exhibit analogous phenomenological trends: supersaturation peaks sharply near the needle outlet, nucleation becomes spatially widespread and intense, while the time for diffusive growth is significantly reduced. Under these conditions, the model predicts smaller mean sizes—trends that parallel the experimental measurements and are consistent with the general framework proposed for flash nano-precipitation systems [61,67].

The simulations highlight that even under high turbulence, local gradients persist shortly downstream. These gradients may explain the residual variability observed experimentally between nominally identical runs, as already reported in similar continuous mixing systems.

The PBE model was calibrated using experimental data obtained at the highest flow-rate condition (36/360 mL/min) and subsequently validated against the intermediate turbulent regime (19/190 mL/min). The kinetic parameters were optimized by minimizing the residual between the predicted mean particle diameter ($d_{76}$) and the experimental Z-average.

For curcumin nanoparticles, the best agreement at 36/360 mL/min was obtained with $A=5\cdot 10^{17}$ [m^−3^s^−1^] and $k_{g}=2.4\cdot 10^{4}$ [nm L g^−1^ s^−1^]. Under these calibration conditions, the model reproduces a mean diameter of 141 nm, consistent with the experimental Z-average (141.0 ± 7.2 nm), corresponding to a relative deviation below 5%. When applied without further adjustment to the 19/190 mL/min condition, the model successfully reproduced the experimentally observed increase in particle size, predicting a diameter of 144 nm, roughly corresponding to the measured value of 148.0 ± 8.3 nm, again corresponding to a deviation below 5% (roughly 3%).

An analogous calibration-validation procedure was applied to liposomes. The optimized parameters were $A=2.7\cdot 10^{19}$ [m^−3^s^−1^] and $k_{g}=4\cdot 10^{2}$ [nm L g^−1^ s^−1^]. At 36/360 mL/min, the model reproduces a mean diameter of 139 nm, fully consistent with the experimental Z-average value (139.0 ± 5.6 nm). At the intermediate flow rate (19/190 mL/min), the model captured the correct increasing trend in particle size, although it slightly underestimated the experimental value, predicting 149 nm compared to 167.0 ± 8.1 nm, corresponding to a deviation of approximately 11%. This discrepancy, limited to a few nanometers, suggests that while the model accurately describes the dominant growth mechanisms and flow-rate dependence, additional effects may become relevant under intermediate turbulent conditions.

A quantitative comparison between model predictions and experimental measurements for both systems and flow conditions is summarized in Table 5.

To provide a more quantitative assessment of model performance, the relative deviation between predicted and experimental mean diameters was evaluated for the investigated conditions. The model shows deviations within approximately 0–5% for the calibration condition and within 3–11% for validation conditions, indicating a satisfactory agreement considering the simplifying assumptions adopted (e.g., absence of aggregation and simplified coupling strategy). These results support the use of the model as a mechanistic tool for interpreting process–structure relationships and for guiding process optimization. Given the limited number of operating conditions and the mechanistic nature of the model, classical statistical indicators such as correlation coefficients were not considered fully representative of model performance.

#### 4.3.3. Integrated Interpretation

Taken together, the combined CFD–PBE framework offers a consistent interpretation of the experimental trends:

- Shorter operational mixing times → faster solvent displacement → earlier, more homogeneous nucleation.
- persistent gradients → extended growth windows → larger and broader particle populations.
- Hydrodynamics modulates the distribution of supersaturation, rather than imposing a single controlling mechanism.

These results reinforce the idea that the coaxial jet mixer primarily acts by tuning the relative timescales of dilution, nucleation and growth—a view compatible with previous discussions on flash nanoprecipitation systems [61,67]. Within this framework, micromixing contributes to setting these timescales, but does not act as an isolated or exclusively controlling mechanism.

These features are also relevant from a process engineering perspective, as they indicate that consistent particle characteristics can be obtained across different operating conditions, supporting the reproducibility of the continuous process.

### 4.4. Discussion

The results collected in the first part of Section 4 delineate a process view of the coaxial jet mixer that goes beyond its use as an investigative device. Taken together, the hydrodynamic mapping, nanoparticle production tests and modeling activities demonstrate that the coaxial configuration can be interpreted as a controllable, continuous production platform, whose performance can be rationally tuned through a limited set of operating parameters.

The first step in this framework is the definition of the fluid-dynamic regimes. The maps constructed using Reynolds number and flow velocity ratio show that the system transitions gradually from laminar focusing to highly entrained jet-like conditions. These maps are not only descriptive: they constitute an operational chart that can be used to select working windows, in the same spirit as design charts used in mixing and reaction engineering. In practice, they indicate where the injected stream remains segregated for long distances and where, instead, rapid dilution can be expected, thereby informing process choices before any formulation is tested.

Overlaying particle formation data on these maps allows the coaxial mixer to be interpreted explicitly as a production process. Both liposomes and curcumin nanoparticles exhibit systematic relationships between operating conditions and product characteristics. Conditions characterized by short operational mixing times produce smaller and more uniform structures, whereas laminar operation tends to lead to broader distributions and reproducibility issues. From a process-development perspective, this means that product attributes can be “navigated” by moving along the hydrodynamic map, rather than by trial-and-error modifications. Importantly, the data also indicate that excessively high flow rates are not automatically beneficial: stability and post-formation aggregation still need to be considered, recalling the typical trade-offs of scalable manufacturing systems.

The modeling results contribute to consolidating this process view. CFD simulations capture the main dilution patterns and reveal how solvent displacement is redistributed when operating conditions are changed. When coupled with the population balance description, the model translates these hydrodynamic fields into expected size trends, showing how earlier and more homogeneous supersaturation favors nucleation-dominated formation pathways. At the same time, the model also exposes present limitations: nucleation kinetics are simplified, stabilization effects are neglected, and gradients at very small scales are only indirectly accounted for. Consequently, the predictive ability of the current framework is primarily qualitative in its current form, while capturing the correct trends and dependencies across operating conditions, providing guidance on tendencies rather than exact quantitative forecasts. Nonetheless, even this level of prediction is valuable at the process-design stage, because it allows rapid screening of feasible operating windows before experimental effort is invested.

It is worth clarifying that the present model includes the coupling between precipitation and solute concentration through the solute mass balance (Equation (18)), where nucleation and growth contribute to solute depletion via the source term $h_{3}$. Therefore, precipitation directly affects the local concentration field and the resulting supersaturation. However, this coupling remains partial, as feedback effects of the dispersed phase on the hydrodynamic field and on transport properties are neglected. In particular, changes in viscosity or density due to particle formation, as well as possible modifications of turbulence and mixing, are not accounted for. These effects are expected to become more relevant in systems with very fast kinetics or at larger scales, where local solute depletion and phase interactions may alter the flow and mixing patterns. Extending the model toward a fully coupled CFD–PBE framework represents a natural direction for future work.

Overall, Section 4 suggests that the coaxial jet mixer should be regarded as a scalable, controllable production technology whose core function is the tuning of characteristic timescales: solvent displacement, supersaturation development and early aggregation. Within this conceptual picture, optimization does not correspond simply to “maximizing turbulence”, but rather to selecting reproducible regions of the map where dilution occurs fast enough to produce the desired structures, while avoiding conditions that may trigger instability or excessive variability. The generality of the approach—demonstrated here on liposomes and curcumin—indicates that the same framework could be extended to other nanoprecipitation systems, provided that formulation-specific stabilization phenomena are adequately incorporated.

From an industrial perspective, indeed, the coaxial jet mixer presents several features that support scalability and process intensification. First, the system operates under continuous flow conditions, enabling steady production with well-defined and reproducible flow rates. For instance, the operating conditions explored in this study (e.g., 36/360 mL/min) already correspond to a throughput on the order of several hundred milliliters per minute, demonstrating the potential for significant productivity compared to batch nanoprecipitation methods. In addition, the process is governed by dimensionless parameters such as Reynolds number and flow velocity ratio, which provide a rational framework for scale-up through hydrodynamic similarity. This suggests that maintaining equivalent flow regimes and mixing times may allow the process to be transferred to larger geometries without altering the underlying particle formation mechanisms. Although a full quantitative assessment of yield and long-term process robustness was beyond the scope of the present study, the consistency of the observed trends across different operating conditions indicates good reproducibility of the system.

There remain, nevertheless, identifiable gaps. A more explicit coupling between hydrodynamics, stabilization mechanisms and long-term particle evolution is still needed; quantitative validation across different chemistries is incomplete; and the transition from laboratory-scale mixers to larger throughputs will require attention to residence-time distributions and equipment design. These open issues, rather than weakening the process concept, outline the natural next steps in developing the coaxial method into a robust manufacturing platform.

## 5. Conclusions

In this work, we developed and critically assessed a coaxial jet antisolvent process for the preparation of both polymeric nanoparticles, using curcumin as a model lipophilic compound, and nanoliposomes. This study combined apparatus design, hydrodynamic characterization, product evaluation, and mechanistic modeling into a single framework aimed at clarifying the relationships among formulation composition, mixing regime, and final particle properties.

At the experimental level, the coaxial configuration proved to be a versatile platform capable of operating under laminar, transitional, and turbulent conditions simply by adjusting the flow rates of solvent and antisolvent streams. Tracking experiments with visual indicators and micromixing probes demonstrated that mixing performance is highly sensitive to flow regime: in laminar conditions, long mixing lengths and slow composition gradients are established, whereas turbulent operation reduces characteristic mixing times to the order of milliseconds. This behavior is central to the precipitation phenomena, because it governs the onset and intensity of supersaturation.

Within this hydrodynamic environment, the coaxial system successfully produced curcumin nanoparticles and nanoliposomes with sizes in the submicron range and with tunable polydispersity. For nanoparticle suspensions, the combination of ethanol solution injection and aqueous antisolvent produced rapid solvent displacement and nucleation. For liposomes, the same device enabled spontaneous lipid self-assembly during solvent exchange, confirming that the coaxial geometry can serve as a unifying technological platform for both polymeric and lipid-based nanocarriers. The overall outcome is a continuous, reproducible process that can, in principle, be adapted to a wide variety of poorly soluble compounds and excipient systems.

To complement these experiments, we constructed a mathematical model coupling hydrodynamics and precipitation. The CFD component, solved under Reynolds-averaged conditions, described the velocity field and the spatial distribution of ethanol–water composition with reasonable agreement with qualitative experimental observations. This allowed us to identify zones of intense shear and rapid dilution, which are expected to coincide with nucleation-dominated regions. On top of this, a first population-balance formulation was implemented, using the method of moments to capture the combined effects of nucleation and growth as functions of local supersaturation. Although still simplified, the model reproduced the qualitative influence of flow regime and solvent composition on the resulting particle size trends.

Taken together, these results indicate that the coaxial jet mixer is not merely a convenient laboratory device but a genuinely informative system in which process variables can be mapped onto physicochemical outcomes with mechanistic clarity. Importantly, the modeling work, even in this initial form, already supports process understanding by providing spatially resolved information that experiments alone cannot easily access.

At the same time, several aspects clearly deserve further development. The current CFD model does not yet incorporate transient effects, potential three-dimensional instabilities, or the feedback of particle formation on local rheology. Likewise, the population balance has been limited to nucleation–growth mechanisms and solved in moment form. Extending the model to the full population balance distribution (PBD) will enable direct prediction of particle size distributions (PSD), rather than only mean sizes or surrogate variables. Incorporating aggregation, breakage, and more detailed solubility kinetics will make the framework more predictive and less reliant on empirical tuning.

Future work should therefore integrate: (i) improved thermodynamic descriptions of solubility and activity coefficients, (ii) detailed nucleation and growth kinetics for different active ingredients, and (iii) validation under continuous, long-term operation and scale-up. Such developments will transform the present model from a qualitative interpretative tool into a quantitative design instrument, capable of guiding mixer geometry, operating windows, and formulation choices a priori.

In conclusion, this study demonstrates that coaxial jet antisolvent mixing represents a promising route for the continuous production of nanocarriers for poorly soluble drugs. By combining experiments with progressively richer modeling, we move toward a rational, science-based methodology for designing nanosized systems with controlled properties. The work presented here constitutes a solid foundation for future refinement and, ultimately, for the translation of coaxial jet precipitation into robust pharmaceutical manufacturing practice.

## Figures and Tables

**Table 1:** Concentration of reagents used for the Villermaux–Dushman reaction.

	Reagent	Concentration [M]
Inner tube	$H_{2}SO_{4}$	0.1
Outer tube	KI	0.035
	$KIO_{3}$	0.007
	$H_{3}BO_{3}$	0.5
	NaOH	0.25

**Table 2:** Main geometric parameters of the coaxial jet mixer used in the simulations.

Parameter	Symbol	Value	Unit
Outer tube length	L	20.5	cm
Outer tube internal diameter	$D_{o}$	3.0	mm
Needle internal diameter (23G)	$d_{i}$	337	μm
Needle external diameter (23G)	$d_{e}$	641.4	μm

**Table 3:** Operating conditions and micromixing characterization for the Villermaux–Dushman experiments performed with the 23G needle. The table reports the flow velocity ratio (FVR), Reynolds number (NRe), identified flow regime, characteristic mixing time $\tau_{\mathrm{mix}}$ obtained from acid–base visualization experiments, and the corresponding segregation index $X_{s}$ measured by spectrophotometric analysis.

FVR	$N_{Re}$	Regime	$\tau_{mix}$ [ms]	$X_{s}$
5	1503	Turbulent	45.91	0.09
7.5	361	Laminar	>170.53	0.38
7.5	820	Laminar	>167.17	0.37
7.5	1082	Transitional	121.00	0.23
7.5	1202	Turbulent	75.00	0.16
7.5	1503	Turbulent	33.93	0.04
7.5	2801	Turbulent	12.85	0.03
10	481	Transitional	218.33	0.51
10	842	Turbulent	96.24	0.05
10	962	Turbulent	62.38	0.05
10	1082	Turbulent	52.67	0.04

**Table 4:** Fluid-dynamic operating conditions employed for nanoparticle production experiments. The table reports the inner and outer flow rates, Reynolds number, flow velocity ratio (FVR), and the corresponding characteristic mixing time $\tau_{\mathrm{mix}}$.

Inner Flow Rate [mL/min]	Outer Flow Rate [mL/min]	$N_{Re}$	FVR	$\tau_{mix}$ [ms]
4.5	45	240	7.5	>1200
19	190	1032	7.5	34
36	360	1920	7.5	13

**Table 5:** Optimized kinetic parameters and comparison between predicted intensity-averaged mean diameter ($d_{76}$) and experimental Z-average values for curcumin nanoparticles and liposomes under different flow conditions. For operating conditions where A and $k_{g}$ are not reported, model results were obtained using the kinetic parameters fitted under the corresponding calibration conditions (^†^), without further adjustment.

System	Flow Rate [mL/min]	A [m^−3^s^−1^]	$k_{g}$ [nm L g^−1^ s^−1^]	$d_{76}$ [nm]	Z-Average [nm]
Curcumin NPs	36/360 ^†^	$5\cdot 10^{17}$	$2.4\cdot 10^{4}$	141	141.0 ± 7.2
Curcumin NPs	19/190	-	-	144	148.0 ± 8.3
Liposomes	36/360 ^†^	$2.7\cdot 10^{19}$	$4\cdot 10^{2}$	139	139.0 ± 5.6
Liposomes	19/190	-	-	149	167.0 ± 8.1

## Data Availability

The data presented in this study are available in this article.
